# Supplementary material for: Ion Substitution-Induced Distorted MOF Lattice with Deviated Energy and Dielectric Properties for Quasi-Solid-State Ion Conductor
Source: Nanomaterials (Basel). 2025 Feb 11;15(4):274. doi: 10.3390/nano15040274 (PMC11857909; doi:10.3390/nano15040274)
Supplement: Supplementary file 1 [file nanomaterials-15-00274-s001.zip › nanomaterials-3416406-supplementary.pdf]

Supplementary Information

Ion Substitution-Induced Distorted MOF Lattice with Deviated Energy and Dielectric Properties for Quasi-Solid-State Ion Conductor

Yike Huang [1], Yun Zheng [1], Yan Guo [1], Qi Zhang [1], Yingying Shen [1], Hebin Zhang [1], Yinan Liu [1], Yihao Zheng [1], Pingshan Jia [1], Rong Chen [1], Lifen Long [1], Zhiyuan Zhang [1], Congcong Zhang [1], Yuanhang Hou [1], Kunye Yan [1], Ziyu Huang [1], Manting Zhang [1], Jiangmin Jiang [1,2], Shengyang Dong [1,3], Wen Lei [1,4] and Huaiyu Shao [1,*]

[1] Joint Key Laboratory of the Ministry of Education, Institute of Applied Physics and Materials Engineering, University of Macau, Avenida da Universidade, Taipa, Macau SAR 999078, China
[2] Jiangsu Province Engineering Laboratory of High Efficient Energy Storage Technology and Equipments, School of Materials Science and Physics, China University of Mining and Technology, Xuzhou 221116, China
[3] Jiangsu Key Laboratory of New Energy Devices & Interface Science, School of Chemistry and Materials Science, Nanjing University of Information Science and Technology, Nanjing 210044, China
[4] The State Key Laboratory of Refractories and Metallurgy, Wuhan University of Science and Technology, Wuhan 430081, China
* Correspondence: hshao@um.edu.mo

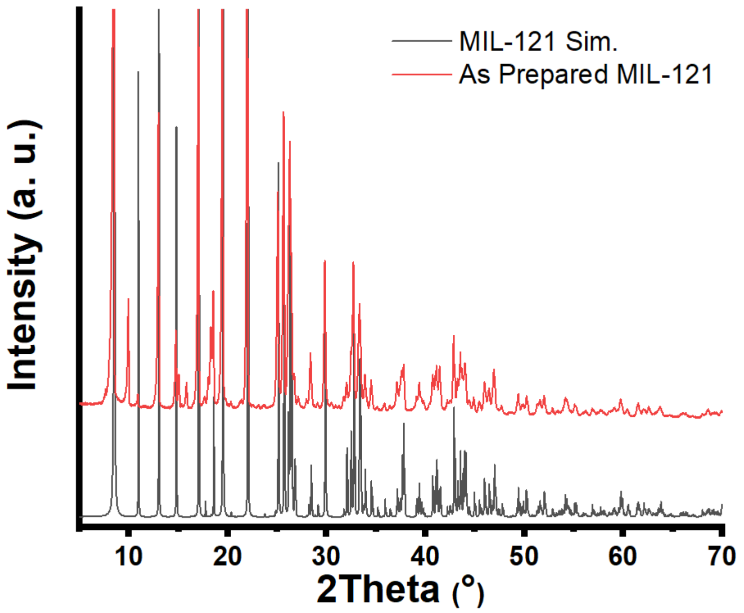

Figure S1. The XRD results of as prepared MOFs and simulated diffraction results.

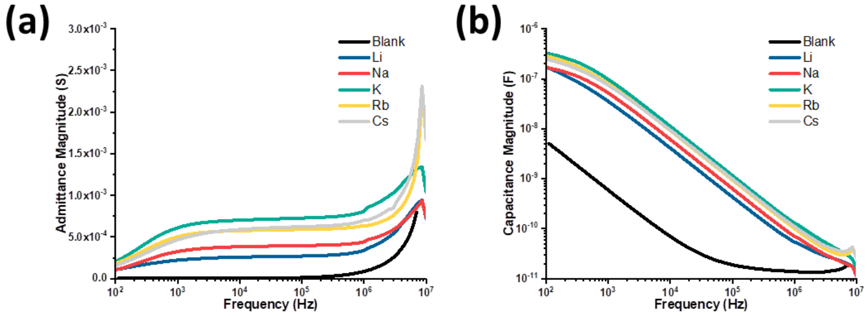

Figure S2. The EIS Bode plots of electrolyte with different ion-substituted MOFs: (a) admittance; (b) capacitance.

Table S1. The element weight ratio of ion-substituted AlBTeC MOFs obtained by ICP-MS.

In wt.%	Al	Li	Na	K	Rb	Cs
AlBTeC300	8.90	0.00	0.15	0.00	0.00	0.00
AlBTeC300-Li	8.66	2.32	0.22	0.00	0.00	0.00
AlBTeC300-Na	9.25	0.00	7.65	0.00	0.00	0.00
AlBTeC300-K	8.82	0.00	0.34	10.52	0.00	0.00
AlBTeC300-Rb	8.14	0.00	0.00	0.00	18.05	0.00
AlBTeC300-Cs	7.79	0.00	0.00	0.00	0.10	23.81

Table S2. The Miller index, diffraction index, and 2θ data of different alkali ion-substituted MOFs.

Miller index	Diffraction index	Calculated	Raw	-Li	-Na	-K	-Rb	-Cs
(110)	(110)	8.51	8.47	8.41	8.44	8.42	8.44	8.42
	(220)	17.10	17.02	16.91	16.96	16.91	16.92	16.91
	(330)	25.77	25.65	25.53	25.58	25.51	25.51	25.49
(100)	(200)	10.99	10.91	10.98	10.83	10.99	10.98	11.13
	(400)	22.03	21.97	22.14	21.77	22.12	22.11	22.39
(010)	(020)	13.03	12.99	12.77	12.99	12.80	12.80	12.66
(001)	(002)	29.13	29.08	29.06	28.92	29.00	29.01	N/A
(11-1)	(22-2)	29.85	29.81	29.66	29.76	29.66	29.64	29.58
(331)	(331)	32.76	32.70	32.61	32.50	32.55	32.58	N/A
(62-1)	(62-1)	33.37	33.32	33.39	33.27	33.47	33.49	33.56
(310)	(620)	35.91	35.85	36.01	35.63	35.95	35.91	N/A
(320)	(640)	42.88	42.84	42.78	42.66	42.75	42.73	42.90
(131)	(262)	54.15	54.16	53.71	53.90	53.61	53.62	N/A

Table S3. The impedance and conductivity data of MOF-based electrolytes.

Samples	R_1 (Ω)	R_2 (Ω)	R_1+R_2 (Ω)	Conductivity (mS cm^{-1})
Raw MOF	-1,265	268,730	267,465	7.8×10^{-3}
-Li	815	2,558	3,373	0.038
-Na	959	1,396	2,355	0.054
-K	704	559	1,263	0.10
-Rb	497	1,042	1,539	0.083
-Cs	388	1,052	1,440	0.088

Table S4. The transference number and its involved data achieved from potentiostatic polarization method.

Samples	I^0 (A)	I^s (A)	R_b^0 (Ω)	R_b^s (Ω)	R_I^0 (Ω)	R_I^s (Ω)	T. Num.
Raw MOF	1.97×10^{-7}	1.35×10^{-8}	117.3	78.7	9.022×10^{4}	9.570×10^{4}	0.096
-Li	3.92×10^{-7}	2.30×10^{-8}	156.3	194.7	2.511×10^{3}	2.347×10^{3}	0.047
-Na	2.73×10^{-7}	3.36×10^{-8}	120.7	111.4	2.253×10^{3}	2.162×10^{3}	0.133
-K	3.15×10^{-7}	4.80×10^{-8}	189.8	177.8	7.913×10^{2}	8.782×10^{2}	0.163
-Rb	1.39×10^{-7}	2.90×10^{-8}	142.9	135.3	6.239×10^{2}	6.668×10^{2}	0.220
-Cs	2.40×10^{-7}	5.20×10^{-8}	144.5	138.8	5.662×10^{2}	5.851×10^{2}	0.226
